# Similar 5-Year Survival in Transfemoral and Transapical TAVI Patients: A Single-Center Experience

**DOI:** 10.3390/bioengineering10020156

**Published:** 2023-01-24

**Authors:** Constantin Mork, Raphael Twerenbold, Brigitta Gahl, Friedrich Eckstein, Raban Jeger, Christoph Kaiser, Oliver Reuthebuch

**Affiliations:** 1 Department of Cardiovascular Research Institute Basel (CRIB), University Hospital Basel, 4031 Basel, Switzerland; 2 Department of Cardiac Surgery, University Hospital Basel, 4031 Basel, Switzerland; 3 University Center of Cardiovascular Science, University Heart and Vascular Center Hamburg, 20251 Hamburg, Germany; 4 Department of Cardiology, University Heart and Vascular Center Hamburg, 20251 Hamburg, Germany; 5 Department of Cardiology, Triemli Hospital Zurich, 8063 Zurich, Switzerland; 6 Department of Cardiology, University Hospital Basel, 4031 Basel, Switzerland

**Keywords:** aortic valve replacement, transcatheter, mortality

## Abstract

Transapical transcatheter aortic valve implantation (TA-TAVI) is generally considered to be associated with increased morbidity and mortality compared with transfemoral transcatheter aortic valve implantation TAVI (TF-TAVI). We aimed to compare different patient risk profiles, access-related complications, and long-term survival using inverse probability treatment weighting. This is a retrospective, single-center analysis of 925 consecutive patients with aortic valve stenosis undergoing TF-TAVI (n = 802) or TA-TAVI (n = 123) at the University Hospital Basel, Switzerland, as a single procedure between September 2011 and August 2020. Baseline characteristics revealed a higher perioperative risk as reflected in the EuroSCORE II (geometric mean 2.3 (95% confidence interval (CI) 2.2 to 2.4) vs. 3.7 (CI 3.1 to 4.5); before inverse probability of treatment weighting (IPTW) *p* < 0.001) in the transfemoral than in the transapical group, respectively. After 30 days, TF-TAVI patients had a higher incidence of any bleeding than TA-TAVI patients (TF-TAVI n = 146 vs. TA-TAVI n = 15; weighted hazard ratio (HR) 0.52 (0.29 to 0.95); *p* = 0.032). After 5 years, all-cause mortality did not differ between the two groups (TF-TAVI n = 162 vs. TA-TAVI n = 45; weighted HR 1.31, (0.92 to 1.88); *p* = 0.138). With regard to our data, we could demonstrate, despite a higher perioperative risk, the short- and long-term safety and efficacy of the transapical approach for TAVI therapies. Though at higher perioperative risk, transapically treated patients suffered from less bleeding or vascular complications than transfemorally treated patients. It is of utmost interest that 5-year mortality did not differ between the groups.

## 1. Introduction

Aortic valve stenosis is the most common valvular heart disease in Europe and North America and its prevalence is steadily increasing due to ageing of the population. Most patients with symptomatic aortic valve stenosis require intervention [1,2]. At an advanced stage of the disease, an intervention is inevitable to ensure survival and improve quality of life. In the past, surgical aortic valve replacement (SAVR) was the gold standard. However, since the first transcatheter aortic valve implantation (TAVI) via an anterograde transseptal approach in 2002 [3], TAVI has since become an established therapy and is now the default therapy in high-risk patients and serves as an alternative to SAVR in intermediate and low-risk patients [4]. In parallel, continuous development in the management and techniques of SAVR has constantly improved the outcome of patients with severe aortic valve stenosis. Nevertheless, the PARTNER trials have proven the efficacy and safety in high- and moderate-risk patients receiving TAVI without the need for cardiopulmonary bypass [5,6,7,8].

Due to continuous improvements in the size and flexibility of delivery systems, the small skin incision and vascular closure devices, as well as increased experience of the interventional cardiologist, transfemoral (TF) access has become the default access route for TAVI [7,9,10]. However, some patients cannot be managed by TF access due to severe calcifications, small vessel caliber, or tortuosity of the peripheral arteries or the aorta. Therefore, several access alternatives have been developed to enable minimally invasive transcatheter treatment for aortic stenosis whenever TF is unfavorable. In 2005, the first case of transapical (TA) TAVI without cardiopulmonary bypass was performed. [9] Since then, the TA approach rapidly developed as the most frequently used alternative access route for patients with unsuitable vascular anatomy [10,11,12].

Patients who underwent TA-TAVIs have been reported to have high rates of 30-day and long-term mortality [13,14]. This observation is aggravated by the high-risk profile of TA-TAVI patients, because they suffer more commonly from coronary artery disease (CAD), peripheral arterial disease (PAD), renal dysfunction, prior cardiac surgery or coronary artery bypass surgery (CABG), and a porcelain aorta [13,14]. Therefore, it is unclear to what extent this increased mortality merely reflects the sicker patient population with an intrinsic higher TA periprocedural risk, or the more invasive TA procedure itself. 

Although alternative access routes have been explored, TA currently is the most frequent alternative access route at the University Hospital Basel, a tertiary care center in cardiovascular medicine. Before each intervention, the patient’s profile is evaluated depending on the risk factors in an interdisciplinary heart team meeting involving cardiac surgeons, interventional cardiologists, anesthesiologists, as well as radiologists. Together, we seek agreement on the best approach based on international guidelines and the patients’ needs and profiles. Even though a thorough evaluation of each patient is done, little is known about the long-term survival rate between TF- and TA-TAVI approaches as well as the long-term complications. With this analysis, we aim to investigate risk factors for outcomes and compare the 5-year survival rate between the TF-TAVI and TA-TAVI approaches using propensity modeling to adjust for baseline variables.

## 2. Material and Methods

All data for this analysis were assessed for the SwissTAVI Registry (date of access 19 October 2020), a national, prospective, multi-center registry in Switzerland focusing on improving the management of patients with aortic valve disease (clinicaltrials.gov NCT01368250). The study was carried out according to the principles of the Declaration of Helsinki and approved by the local ethics committees (ethics committee in Basel (reference number 305/11; 2011; since then permission through the ethics committee Bern, project ID: 2021-01738). The SwissTAVI Registry uses for data collection a web-based database (www.swisstavi.ch) with standardized case report forms at all centers performing TAVI in Switzerland. The first patient receiving the TAVI procedure was recruited in September 2011. The data of all participating centers are collected at baseline and during follow up, which is performed according to a prespecified protocol. All clinical events are prospectively collected and adjudicated by a dedicated clinical event committee according to the standardized criteria of the Valve Academic Research Consortium 2 (VARC-2) [15]. The study protocol of the SwissTAVI registry was approved by the institutional review board of all participating sites and by the local cantonal ethics committee. Written informed consent was obtained from all patients. The authors designed the study, gathered, and analyzed the data.

### 2.1. Study Population

**Figure 1:**
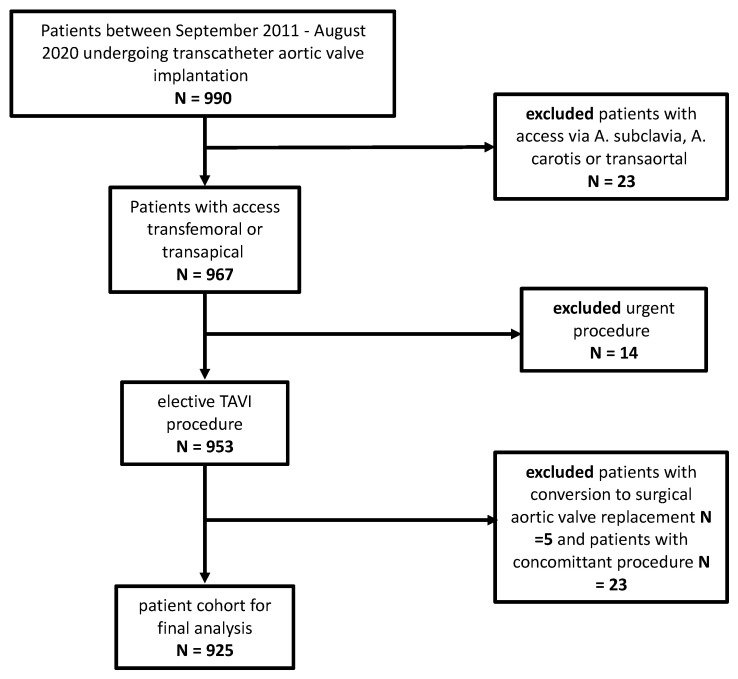
Flowchart.

Data of 990 consecutive patients with aortic valve stenosis undergoing TAVI procedures at the University Hospital of Basel, Switzerland, between September 2011 and August 2020, were extracted from the SwissTAVI Registry. Patients with concomitant procedures or other vascular access (via subclavian artery or transaortic) were excluded, leaving a study sample of 925 patients. (Figure 1) The most common indication in both TAVI groups was a severe stenosis of the native aortic valve, while 22 patients (3%) in the TF-TAVI group suffered from severe stenosis of a biological aortic prosthesis, and one patient from a combined severe regurgitation and stenosis of a biological aortic valve prosthesis. Among the TA-TAVI patients, one patient each had a severe stenosis of a biological valve, a severe regurgitation of a biological or a degenerative previous TAVI prosthesis.

### 2.2. Pre-Interventional Diagnostics

All patients with severe aortic stenosis considered for an intervention underwent coronary angiography, transthoracic echocardiography, and three-dimensional cardiovascular computer tomography (CT). After evaluation, the patient’s profile was evaluated depending on the risk factors in an interdisciplinary heart team meeting involving cardiac surgeons, interventional cardiologists, anesthesiologists, as well as radiologists. The CT scan was carefully analyzed using the 3-mensio planning software (3mensio Medical Imaging, Bilthoven, The Netherlands) to determine valvular specifications, such as annular size, degree of valve calcification, distance to coronary ostia, and implantation angle. Vascular entities were analyzed with regard to diameter and characteristics of the iliofemoral and aortic axis. In case of a small peripheral vessel access, severe annular calcification, abdominal aortic aneurysm or vessel tortuosity, TA was considered to be the best option. The TAVI procedure was performed in the cardiac catheterization lab (TF) or hybrid operating room (TA) with an interdisciplinary team of cardiac surgeons, interventional cardiologists, anesthesiologists supported by scrub nurses and radiological technologists. 

### 2.3. TAVI Devices

Over the years, several different devices with different generations were implanted in our center. All percentage distributions of implanted TAVIs are listed in Table S1 in Supplementary Materials. The most common devices for TF-TAVI were St. Jude Medical Portico^TM^ (22%) (St. Jude Medical, Saint Paul, MN, USA), Edwards Sapien^TM^ 3 (19%), Sapien^TM^ XT (8.6%), (Edwards Lifesciences, Irvine, CA, USA), Medtronic CoreValve (8.6%), Boston Scientific Lotus^TM^ (8.5%), Medtronic Evolut R^TM^ (7.5%), Medtronic Evolut Pro^TM^ (4.7%) (Medtronic Inc., Minneapolis, MN, USA), and Lotus Edge^TM^ (2.4%) (Boston Scientific, Marlborough, MA, USA)

In case of TA-TAVI, the most commonly used valves were the JenaValve (46%) (JenaValve Technology, Inc., Munich, Germany) and the Edwards Sapien^TM^ 3 (43%) (Edwards Lifesciences, Irvine, CA, USA).

### 2.4. Study End Points 

The primary endpoint of this study was all-cause mortality at 5 years in patients undergoing either TF- or TA-TAVI. The secondary endpoints included outcomes at 30 days according to the VARC-2 definitions, which include major adverse cardiac and cerebrovascular events such as death, myocardial infarction, stroke, bleeding, and aortic valve re-intervention [15].

### 2.5. Statistical Analysis

To investigate to what degree the outcome might be associated with the treatment, we used propensity modeling to achieve balanced treatment groups with respect to risk factors, given the observational origin of the data. We used inverse probability of treatment weighting (IPTW) to calculate average treatment effects. The propensity score with the kernel density (Figure S1 in Supplementary Materials) and the standardized differences before and after IPTW (Figure S2 in Supplementary Materials) are mentioned in the Supplementary Materials. We included age, sex, Society of Thoracic Surgeons (STS) score (after log transformation), chronic obstructive pulmonary disease (COPD), CAD, PAD, atrial fibrillation, hypertension, body surface area, and diabetes as covariates into the propensity model. We censored treatment weights exceeding the 1st and 99th percentiles [16] and calculated standardized differences for each variable to assess residual imbalances between the groups, using the formulae proposed in Austin et al. [17]. Standardized differences of ±0.1 standard deviation units or less are considered to indicate irrelevant difference, ±0.2 might still be considered acceptable. We analyzed our primary outcome survival in a time-to-event manner after IPTW, including a 30-day landmark to balance risk differences related to the procedure. We plotted Kaplan–Meier curves for visualization and calculated hazard ratios using Cox regression, with results are referred to as “weighted hazard ratio”. Proportional hazard assumptions were checked using Schoenfeld residuals.

Continuous variables were presented as mean ± standard deviation if normally distributed, or as geometric mean if distribution was skewed, with standard deviations back transformed from the log scale. Corresponding *p*-values were calculated using linear regression on the variable or on the log transformed variable, respectively. Categories were presented as numbers and percentages, *p*-values were calculated using logistic regression for binary variables or multinomial regression else. After IPTW, all *p* values and confidence intervals were based on robust standard deviations. Statistical analyses were performed using Stata 16 (StataCorp LLC, College Station, TX, USA).

## 3. Results

### 3.1. Patient’s Characteristics

From September 2011 to August 2020, a total of 990 documented patients were eligible for this analysis. In addition, 71% of the patients were octogenarians (mean 82 ± 7 years). A total of 925 patients received either a TF-TAVI or TA-TAVI (TF-TAVI: n = 802 (86.7%) vs. TA-TAVI: n = 123 (13.3%)) as a single procedure. Patients with a TA approach had a higher prevalence of COPD, stroke, history of cardiac surgery, previous CABG, PAD, and suffered more often from arterial hypertension, syncope, as well as diabetes mellitus (Table 1), whereas patients with TF-TAVI more often had previous valve surgery and atrial fibrillation. Overall, TA-TAVI patients had a higher operative risk as reflected in the EuroSCORE II value (TF-TAVI: geometric mean 2.3 (95% confidence interval (CI) 2.2 to 2.4) vs. TA-TAVI: 3.7 (CI 3.1 to 4.5); before inverse probability of treatment weighting (IPTW) *p* < 0.001) and STS score (TF-TAVI: 4.0 (CI 3.8 to 4.1) vs. TA-TAVI 5.4 (CI 4.8 to 6.0); before IPTW *p* < 0.001). Overall, no significant difference in preoperative anti-platelet and anti-coagulation therapy was seen, except for Aspirin, with a higher medication in the TA-TAVI group TF_TAVI: n = 433 (54%) vs. TA-TAVI n = 90 (73%); *p* < 0.001). (Table S3 in Supplementary Materials).

**Figure 2:**
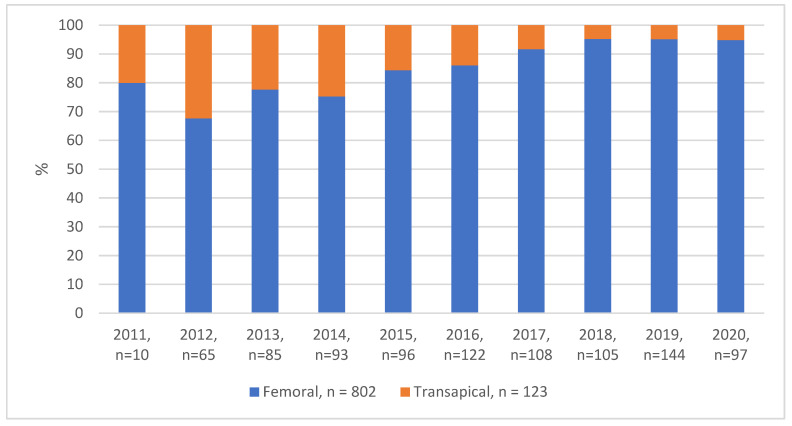
Percentage distribution of transfemoral and transapical TAVI per year: Shown is the percentage distribution for both procedures in each year. At the beginning of 2015, there is a trend in favor of using transfemoral access and a decrease of using transapical access for TAVI each year. Since 2018, around 5% of patients per year undergo transapical approach.

Over the last decade, there has been a noted shift in the choice of access route—while TF as an access route increased over time, there was a decrease in TA-TAVIs (Figure 2). In case of TF-TAVI, 90% (n = 723) of the patients had local anesthesia and 10% (n = 78) had general anesthesia. All TA-TAVI patients (n = 123 (100%)) received general anesthesia. In the TF-TAVI group, procedural time (68 ± 26 min vs. 86 ± 43 min; before IPTW *p* < 0.001) was shorter and less radiocontrast agent was used as compared to TA-TAVI (190 ± 73 mL vs. 154 ± 78 mL, *p* < 0.001). The main access site in the TF-TAVI group was predominantly treated with preclosure devices (n = 787, 98%). In the TA-TAVI group, the main access site (apex) was closed with purse-string sutures (n = 123 (100%)) (Table 2). The detailed numerical distribution per year is in Table S2 in the Supplementary Materials.

### 3.2. Procedural Complication and Early Procedural Outcomes

Overall, both groups had a low incidence of procedural complications (Table 3). The two groups (TF-TAVI n = 24, (3%) vs. TA-TAVI n = 2, (2%); after IPTW *p* = 0.454) showed a low incidence of aortic valve dislocation. Only three cases were reported within the group of TF-TAVI that required conversion to another access route, either due to massive, unexpected calcification, tortuosity, or undersized diameter of the femoral artery. In 6% of the TF-TAVI patients (n = 46), an access vessel complication was reported and in 2% (n = 17), a vascular surgeon had to intervene for an open vascular surgical repair. None of these complications occurred in the TA-TAVI group. At discharge, aortic regurgitation grades were measured via trans esophageal echocardiography and were similar in both groups (*p* = 0.646). In both groups, more than 50% did not have aortic regurgitation at all (TF-TAVI n = 454 (57%), TA-TAVI n = 64 (52%)) and approximately 40% had mild aortic regurgitation (TF-TAVI n = 325 (41%), TA-TAVI n = 57 (46%)). The mean gradient above the prosthetic aortic valve was comparably low at discharge in both groups (TF-TAVI 8mmHg (CI 8 to 9 mmHg) vs. TA-TAVI 11 mmHg (CI 10 to 12 mmHg); after IPTW *p* = 0.007). Patients who underwent the transapical approach stayed slightly longer in the intensive care unit (TF-TAVI 2 days (CI 2 to 2 days) vs. TA-TAVI 2 days (CI 2 to 3 days); after IPTW *p* = 0.019), however longer on the general ward (TF-TAVI 5 days (CI 5 to 6 days) vs. TA-TAVI 9 days (CI 8 to 10 days); after IPTW *p* < 0.001). 

According to the VARC-2 criteria, bleeding and vascular complications within 30 days after intervention were seen more often after TF-TAVI than after TA-TAVI, corresponding to an IPT weighted hazard ratio of 0.52, 95% CI 0.29 to 0.95, *p* = 0.032, and 0.18, CI 0.07 to 0.42, *p* < 0.001, respectively. (Table 4) We did not see a difference regarding 30-day mortality, weighted hazard ratio (HR) 1.11, CI 0.43 to 2.87, *p* = 0.82, nor regarding any other pre-specified outcome. 

**Figure 3:**
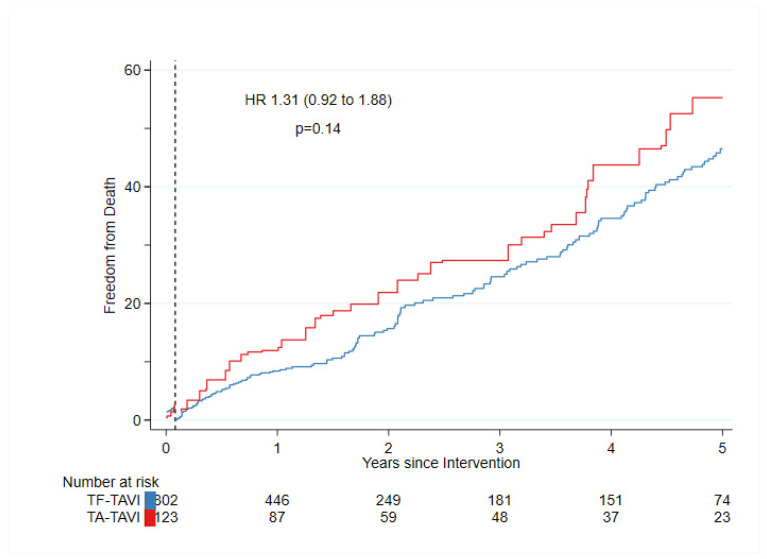
Mortality in patients who underwent TA-TAVI was similar to patients who underwent TF-TAVI after IPTW with a landmark inserted at 30 days after the procedure.

More patients needed a permanent pacemaker after TF-TAVI than after TA-TAVI, (weighted HR 0.22, CI 0.09 to 0.53, *p* = 0.001) due to a difference in valve selection and a substantially higher incidence of a high-grade atrioventricular blockage. Figure 3 shows IPT-weighted 30 days’ landmark estimates for freedom from death. A crude (before IPTW) Kaplan-Meier curve with confidence bands which overlap is in Figure S3 in Supplementary Materials.

### 3.3. 5-Year Follow-Up

The results of the long-term follow-up of patients after TF- and TA-TAVI are shown in Table 4. We did not find an association of treatment and death (weighted HR TF/TA TAVI: 0–5 years after IPTW; 1.31 (0.92 to 1.88); *p* = 0.138). Similarly, upon calculating the HR between both intervention groups, no favor for one or the other intervention in outcomes, such as stroke, myocardial infarction (MI), acute kidney injury or life-threatening bleeding, major bleeding, structural valve deterioration, or reintervention, was observed (Table 4). Minor bleeding was slightly lower after TA TAVI (weighted HR TF/TA TAVI: 0–5 years after IPTW; 0.13 (0.04 to 0.48); *p* ≤ 0.001).

## 4. Discussion

Ever since the introduction of transcatheter aortic valve implantation, the two main access routes are transfemoral and transapical. As reported from other centers [18], a constant shift to the TF-TAVI was also visible at our institute (Figure 2). The improved accuracy of computed tomography and planning software, the decreased sheath size and increased flexibility of the TF-TAVI systems, no need for general anesthesia or post-interventional pleural drainage, are beneficial. As a result, the majority of patients qualify for a TF-TAVI. This stimulates a widespread controversial discussion of TA-TAVI being a high-risk intervention. Nevertheless, due to ongoing ageing of the population and increasing numbers of severe PAD, the TF-TAVI might not be the favorable route of access due to the lack of sufficient vessel diameter in the ilio-femoral artery or aorta, and the risk for dissection, rupture, or thrombosis. Regarding our single center study, we can report seven major findings. 

First, patients in the TA-TAVI group had a higher perioperative risk due to differences in baseline characteristics. However, even though the group of TA-TAVI reflected higher risk patients, the primary and secondary outcomes did not differ between TF- and TA-TAVI. Therefore, TA-TAVI should be always considered as a safe alternative access with a similar survival rate. Similar results were reported by a prospective single center study with 1000 patients by Schymik, G et al. [19], which supports our findings. Second, the scarce need for intraoperative conversion to another access route reflects a concise treatment selection for each patient within the interdisciplinary heart team. This is even lower compared to rates reported by the SENTINAL and SOURCE registries (TF 4.7% and 1.7%; TA 3.2% and 3.5%, respectively) [20,21]; Third, even though the incidence was low, the TF-TAVI cohort had a higher incidence of bleeding complications during the procedure. Major bleeding and vascular complications occur significantly more often in TF-TAVI as already reported by previous studies [21,22,23,24]. However, the low incidence of access bleeding in the current analysis might be due to the use of an ultrasound-driven puncture of the main access (the femoral artery) to reduce the risk of puncturing the false vessel or calcified area of the access vessel. Fourth, permanent pacemaker implantation rates were low (15%) in the overall group compared to data from a large multicenter collaborative study [22]. However, they were four times higher in TF-TAVI than TA-TAVI patients. Most of the patients received a dual chamber pacemaker due to a higher grade of atrioventricular block. In contrast to these findings, the propensity matched analysis in the PARTNER TRIAL I showed no such differences in both groups [10]. We assume the use of prosthesis with low pacemaker rates for TA-TAVI procedures, such as the JenaValve (JenaValve Technology GmbH, Munich, Germany) causative for this [25]. Fifth, 30-day mortality after propensity modeling was similar for both groups. In our center, we were able to achieve lower mortality rates in comparison to other trials, such as the SOURCE, UK TAVI, PRAGMATIC, and complete SwissTAVI, which range from 3.6% to 6.4% for TF-TAVI and 9.5% to 15.7% for TA-TAVI [21,22,23,24]. Considering the baseline characteristics, the patients’ factors are unlikely to be responsible for this difference. We consider the improvement of the delivery and valve systems as well as the increasing operator experience causal [26,27]. Sixth, TA-TAVI were all performed in the modern Hybrid operating room with the newest imaging solution (ARTIS pheno®, Siemens Healthineers) and TA-TAVI patients were as well under a cardiac anesthesiologist’s care during the implantation with the broad spectrum of treatment options. Seventh, upon evaluation of the long-term outcomes, we did not find an association of treatment and death and there were no significant differences in the occurrence of stroke, acute renal injury, or MI.

Our findings validate and broaden previous studies regarding the understanding and importance of meticulous evaluation of the heart to establish the best possible treatment. With this, we can ensure a safe access route and comparable long term survival rate for both patient’s groups. Despite findings from previous literature, we could show convincing results indicating that TA is an appropriate therapy and can be used successfully as a bail-out strategy for TF in an unfavorable or even hostile environment.

Some limitations should be considered when interpreting these findings. First, it is a single-center study and there is a lack of random assignment to treatment groups due to the heart team´s decisions. Second, from the beginning of the study until this retrospective analysis, there has been major technical development, especially in the sense of transfemoral delivery systems. With the latest delivery systems, smaller and more precise vascular access has become feasible and procedure-related bleedings may be more preventable. Third, as can be seen from the standardized differences, propensity modeling did not achieve fully balanced treatment groups with respect to the risk of outcomes, which indicates that there might be some residual confounding factors.

## 5. Conclusions

In this single-center observational study involving 925 consecutive patients with aortic valve stenosis undergoing TF- or TA-TAVI, we demonstrated compared short- and long-term safety of both treatment groups. Major vascular complications and indications for permanent pacemaker implantation were higher in the TF-TAVI group. The present study shows the importance of the interdisciplinary heart team to talk over the best individual patient treatment option. In contrast to previous literature and despite the ongoing shift to TF-TAVI procedures, the present study shows that TA-TAVI is a safe and efficacious treatment and is an adequate alternative to TF-TAVI.

## Figures and Tables

**Table 1:** Baseline characteristics before and after IPTW.

	Before IPTW				After IPTW			
	TF-TAVI n = 802	TA-TAVI n = 123	SD	*p*	TF-TAVI n = 802	TA-TAVI n = 123	SD	*p*
Age (years)	82 ± 6	82 ± 7	−0.110	0.235	82 ± 6	82 ± 6	0.008	0.933
Body mass index (kg/m2)	27 ± 5	26 ± 5	−0.242	0.014	27 ± 5	27 ± 7	0.012	0.908
Euro SCORE II Value	2.3 (2.2 to 2.4)	3.7 (3.1 to 4.5)	0.641	<0.001	2.4 (2.2 to 2.5)	3.3 (2.7 to 4.1)	0.487	0.047
STS risk score	4.0 (3.8 to 4.1)	5.4 (4.8 to 6.0)	0.689	<0.001	4.1 (3.9 to 4.3)	4.6 (4.0 to 5.3)	0.243	0.243
LVEF (%)	52 (51 to 53)	53 (50 to 55)	0.764	0.712	51 (50 to 53)	52 (49 to 56)	0.735	0.678
Female sex – no. (%)	432 (54%)	50 (41%)	0.267	0.007	416 (52%)	55 (44%)	0.150	0.178
Diabetes mellitus – no. (%)	236 (29%)	34 (28%)	0.040	0.685	236 (29%)	35 (29%)	0.015	0.892
Arterial hypertension – no. (%)	649 (81%)	106 (86%)	−0.142	0.163	656 (82%)	107 (87%)	−0.152	0.172
Dyslipidemia – no. (%)	467 (58%)	84 (68%)	−0.210	0.035	473 (59%)	80 (65%)	−0.132	0.243
COPD – no. (%)	71 (9%)	32 (26%)	−0.464	<0.001	88 (11%)	20 (16%)	−0.150	0.117
History of Stroke – no. (%)	102 (13%)	17 (14%)	−0.033	0.734	105 (13%)	18 (15%)	−0.054	0.620
Previous aortic valvuloplasty – no. (%)	29 (4%)	3 (2%)	0.069	0.509	30 (4%)	3 (2%)	0.073	0.547
Coronary artery disease – no. (%)	440 (55%)	91 (74%)	−0.408	<0.001	461 (57%)	83 (67%)	−0.204	0.081
History of PCI – no. (%)	276 (34%)	60 (49%)	−0.295	0.002	291 (36%)	52 (42%)	−0.115	0.288
History of MI – no. (%)	123 (15%)	26 (21%)	−0.151	0.105	131 (16%)	22 (18%)	−0.037	0.723
Atrial fibrillation – no. (%)	166 (21%)	13 (11%)	0.282	0.010	155 (19%)	14 (12%)	0.211	0.082
Peripheral artery disease	103 (13%)	52 (42%)	−0.698	<0.001	133 (17%)	29 (23%)	−0.165	0.072
Previous cardiac surgery	84 (10%)	23 (19%)	−0.235	0.009	91 (11%)	19 (15%)	−0.113	0.257
Previous CABG – no. (%)	64 (8%)	23 (19%)	−0.319	<0.001	71 (9%)	19 (15%)	−0.196	0.043
Previous valve surgery – no. (%)	31 (4%)	3 (2%)	0.082	0.438	32 (4%)	1 (1%)	0.178	0.078
Syncope – no. (%)	63 (8%)	17 (14%)	−0.195	0.029	63 (8%)	20 (16%)	−0.256	0.012
NYHA (III or IV) – no. (%)	429 (54%)	75 (61%)	−0.154	0.118	430 (54%)	72 (59%)	−0.101	0.369
Indication				0.365				<0.001
severe stenosis of native valve	779 (97%)	120 (98%)	0.027		778 (97%)	122 (99%)	0.128	
severe stenosis of bioprosthesis	22 (3%)	1 (1%)	−0.146		23 (3%)	0 (0%)	−0.207	
severe regurgitation of an aortic bioprosthesis	1 (0%)	1 (1%)	0.101		1 (0%)	0 (0%)	0.013	
degenerative transcatheter heart valve prosthesis	0 (0%)	1 (1%)	0.128		0 (0%)	1 (1%)	0.120	

IPTW: inverse probability of treatment weighting; TF-TAVI: transfemoral transcatheter aortic valve implantation; TA-TAVI: transapical transcatheter aortic valve implantation; SD: standard deviation; STS: Society of Thoracic Surgeons; LVEF: left ventricular ejection fraction; COPD: chronic obstructive lung disease; PCI: percutaneous coronary intervention; MI: myocardial infarction; CABG: coronary artery bypass graft; NYHA: New York Heart Association.3.2. Procedural Details.

**Table 2:** Procedural details.

	Before IPTW				After IPTW			
Procedural Details	TF-TAVI n = 802	TA-TAVI n = 123	SD	*p*	TF-TAVI n = 802	TA-TAVI n = 123	SD	*p*
Procedure time – min	68 ± 26	86 ± 43	0.532	<0.001	67 ± 26	85 ± 50	0.434	<0.001
Total contrast administered – ml	190 ± 73	154 ± 78	−0.473	<0.001	190 ± 76	156 ± 91	−0.400	<0.001
Valve size – mm	27 (27 to 27)	25 (25 to 26)	0.862	<0.001	27 (27 to 27)	25 (25 to 26)	0.858	<0.001
Balloon valvuloplasty – no. (%)	680 (85%)	113 (92%)	−0.222	0.040	682 (85%)	114 (93%)	−0.253	0.042
Device closure of femoral artery	788 (98%)	41 (34%)	1.864	<0.001	787 (98%)	37 (30%)	2.021	<0.001
Number of valves used				0.672				<0.001
1	777 (97%)	118 (96%)	−0.052		776 (97%)	118 (96%)	−0.029	
2	21 (3%)	5 (4%)	0.081		22 (3%)	5 (4%)	0.057	
3	4 (1%)	0 (0%)	−0.100		4 (0%)	0 (0%)	−0.099	

IPTW: inverse probability of treatment weighting; TF-TAVI: transfemoral transcatheter aortic valve implantation; TA-TAVI: transapical transcatheter aortic valve implantation; SD: standard deviation.

**Table 3:** Procedural complication and early outcome.

	Before IPTW				After IPTW			
Procedural Complication and Early Outcome	TF-TAVI n = 802	TA-TAVI n = 123	SD	*p*	TF-TAVI n = 802	TA-TAVI n = 123	SD	*p*
Reposition with snare	6 (1%)	0 (0%)	0.123	1.000	6 (1%)	0 (0%)	0.123	1.000
Valve in valve	18 (2%)	4 (3%)	−0.06	0.497	18 (2%)	3 (3%)	−0.029	0.753
Valve retrieval	12 (1%)	0 (0%)	0.174	0.385	13 (2%)	0 (0%)	0.182	0.385
Valve dislocation	23 (3%)	2 (2%)	0.084	0.435	24 (3%)	2 (2%)	0.086	0.454
Conversion to transapical approach	3 (0%)	0 (0%)	0.087	1.000	3 (0%)	0 (0%)	0.084	1.000
Cardiac tamponade or rupture	7 (1%)	1 (1%)	0.007	0.947	8 (1%)	1 (1%)	0.041	0.659
Hemodynamic instability requiring treatment	29 (4%)	3 (2%)	0.069	0.509	30 (4%)	2 (1%)	0.150	0.104
Resuscitation	14 (2%)	1 (1%)	0.065	0.584	14 (2%)	1 (0%)	0.121	0.210
Femoral artery complication	44 (5%)	0 (0%)	0.341	0.002	46 (6%)	0 (0%)	0.347	0.002
- Stenosis	6 (1%)	0 (0%)	0.123	1.000	7 (1%)	0 (0%)	0.128	1.000
- Rupture	2 (0%)	0 (0%)	0.071	1.000	3 (0%)	0 (0%)	0.085	1.000
- Hematoma (>2 units transfusion)	4 (0%)	0 (0%)	0.100	1.000	4 (1%)	0 (0%)	0.101	1.000
- Stent placed	11 (1%)	0 (0%)	0.167	0.377	11 (1%)	0 (0%)	0.168	0.377
- Need for vascular surgery	17 (2%)	0 (0%)	0.208	0.150	17 (2%)	0 (0%)	0.211	0.150
Coronary artery occlusion	1 (0%)	0 (0%)	0.050	1.000	1 (0%)	0 (0%)	0.049	1.000
Annulus rupture/aortic dissection	4 (0%)	0 (0%)	0.100	1.000	5 (1%)	0 (0%)	0.113	1.000
Type of femoral artery complication				1.000				<0.001
- dissection	15 (2%)	0 (0%)	−0.19		16 (2%)	0 (0%)	−0.203	
- perforation	13 (2%)	0 (0%)	−0.18		12 (2%)	0 (0%)	−0.176	
- occlusion	13 (2%)	0 (0%)	−0.18		14 (2%)	0 (0%)	−0.184	
Aortic valve peak gradient (mmHg)Peak gradient (mmHg)	15 (15 to 16)	19 (17 to 21)	0.705	0.003	15 (15 to 16)	19 (17 to 21)	0.691	0.003
Aortic valve mean gradient (mmHg)	8 (8 to 9)	11 (9 to 12)	0.690	0.015	8 (8 to 9)	11 (10 to 12)	0.698	0.007
Aortic regurgitation grade postmeasure/discharge				0.667				0.646
- none	453 (57%)	63 (51%)	−0.106		454 (57%)	64 (52%)	−0.089	
- mild	327 (41%)	56 (46%)	0.096		325 (41%)	57 (46%)	0.113	
- moderate	19 (2%)	3 (2%)	0.004		19 (2%)	2 (1%)	−0.084	
- severe	3 (0%)	1 (1%)	0.057		4 (0%)	0 (0%)	−0.010	
Intensive care unit – days	2 (2 to 2)	2 (2 to 3)	0.678	0.005	2 (2 to 2)	2 (2 to 3)	0.653	0.019
General ward - days	5 (5 to 6)	9 (8 to 10)	0.860	<0.001	5 (5 to 6)	9 (8 to 10)	0.877	<0.001

IPTW: inverse probability of treatment weighting; TF-TAVI: transfemoral transcatheter aortic valve implantation; TA-TAVI: transapical transcatheter aortic valve implantation; SD: standard deviation.3.4. 30-Day Follow-Up.

**Table 4:** Short and long-term follow up (landmark 30 days).

				Before IPTW		After IPTW	
Outcome	Time Interval	TF-TAVI n = 802	TA-TAVI n = 123	HR	*p*	HR	*p*
Death	≤30 d	17	7	2.67 (1.11 to 6.45)	0.028	1.11 (0.43 to 2.87)	0.834
	30 d-5 y	145	38	1.18 (0.83 to 1.69)	0.354	1.33 (0.91 to 1.95)	0.141
	0-5 y	162	45	1.31 (0.94 to 1.82)	0.115	1.31 (0.92 to 1.88)	0.138
	Overall	164 (20%)	47 (38%)				
Stroke	≤30 d	30	7	1.52 (0.67 to 3.47)	0.315	1.11 (0.42 to 2.92)	0.832
	30 d-5 y	17	2	0.59 (0.14 to 2.58)	0.488	1.01 (0.21 to 4.81)	0.987
	0-5 y	47	9	1.14 (0.56 to 2.33)	0.715	1.07 (0.47 to 2.47)	0.867
	Overall	47 (6%)	10 (8%)				
MI	≤30 d	10	2	1.30 (0.29 to 5.94)	0.733	0.43 (0.09 to 2.06)	0.294
	30 d-5 y	11	3	1.25 (0.35 to 4.49)	0.735	1.09 (0.27 to 4.41)	0.900
	0-5 y	21	5	1.27 (0.48 to 3.38)	0.633	0.82 (0.27 to 2.42)	0.713
	Overall	21 (3%)	6 (5%)				
AKI	≤30 d	15	4	1.73 (0.57 to 5.20)	0.332	1.14 (0.35 to 3.69)	0.828
	30 d-5 y	1	1	5.77 (0.36 to 92.3)	0.215	3.78 (0.24 to 60.2)	0.347
	0-5 y	16	5	2.00 (0.73 to 5.46)	0.176	1.55 (0.51 to 4.71)	0.439
	Overall	16 (2%)	5 (4%)				
Any bleeding	≤30 d	146	15	0.65 (0.38 to 1.11)	0.114	0.52 (0.29 to 0.95)	0.032
	30 d-5 y	26	5	0.88 (0.34 to 2.29)	0.787	0.62 (0.20 to 1.93)	0.407
	0-5 y	172	20	0.69 (0.44 to 1.10)	0.124	0.54 (0.32 to 0.92)	0.022
	Overall	172 (21%)	20 (16%)				
Life threatening bleeding	≤30 d	15	5	2.17 (0.79 to 5.96)	0.134	1.75 (0.56 to 5.50)	0.339
	30 d-5 y	13	4	1.49 (0.48 to 4.58)	0.489	1.22 (0.34 to 4.35)	0.761
	0-5 y	28	9	1.81 (0.85 to 3.86)	0.121	1.47 (0.62 to 3.48)	0.377
	Overall	28 (3%)	9 (7%)				
Major bleeding	≤30 d	54	7	0.84 (0.38 to 1.84)	0.659	0.77 (0.32 to 1.85)	0.562
	30 d-5 y	4	0	no event	1.00	no event	1.00
	0-5 y	58	7	0.77 (0.35 to 1.70)	0.523	0.71 (0.30 to 1.70)	0.444
	Overall	58 (7%)	7 (6%)				
Minor bleeding	≤30 d	77	3	0.25 (0.08 to 0.79)	0.018	0.13 (0.04 to 0.48)	0.002
	30 d-5 y	9	1	0.50 (0.06 to 3.95)	0.511	0.14 (0.02 to 1.07)	0.058
	0-5 y	86	4	0.29 (0.10 to 0.78)	0.014	0.13 (0.04 to 0.41)	<0.001
	Overall	86 (11%)	4 (3%)				
Vascular bleeding	≤30 d	137	6	0.28 (0.12 to 0.64)	0.002	0.18 (0.07 to 0.42)	<0.001
	30 d-5 y	2	0	no event	1.000	no event	
	0-5 y	139	6	0.28 (0.12 to 0.63)	0.002	0.17 (0.07 to 0.41)	<0.001
	Overall	139 (17%)	6 (5%)				
SVD	≤30 d	3	1	2.17 (0.23 to 20.87)	0.502	4.23 (0.45 to 39.8)	0.207
	30 d-5 y	20	3	0.72 (0.21 to 2.42)	0.590	0.62 (0.16 to 2.39)	0.485
	0-5 y	23	4	0.87 (0.30 to 2.51)	0.792	0.99 (0.29 to 3.34)	0.986
	Overall	24 (3%)	4 (3%)				
Reintervention	≤30 d	13	2	1.00 (0.23 to 4.44)	0.998	0.85 (0.19 to 3.80)	0.831
	30 d-5 y	9	2	1.09 (0.24 to 5.09)	0.908	1.49 (0.25 to 8.93)	0.661
	0-5 y	22	4	1.04 (0.36 to 3.04)	0.936	1.13 (0.35 to 3.67)	0.841
	Overall	22 (3%)	5 (4%)				
PM	≤30 d	169	7	0.25 (0.12 to 0.54)	<0.001	0.22 (0.09 to 0.53)	0.001
	30 d-5 y	12	3	1.05 (0.30 to 3.73)	0.941	1.17 (0.33 to 4.12)	0.803
	0-5 y	181	10	0.33 (0.17 to 0.62)	0.001	0.31 (0.15 to 0.62)	0.001
	Overall	181 (23%)	10 (8%)				

IPTW: inverse probability of treatment weighting; TF-TAVI: transfemoral transcatheter aortic valve implantation; TA-TAVI: transapical transcatheter aortic valve implantation; HR: hazard ratio; MI: myocardial infarction; AKI: acute kidney injury; SVD: structural valve deterioration; PM: pacemaker.

## Data Availability

All data generated and analyzed during this study are included in this study published article (and its Supplementary Materials).

## Supplementary Materials

The following supporting information can be downloaded at: https://www.mdpi.com/article/10.3390/bioengineering10020156/s1, Figure S1: Propensity Score, kernel density; Figure S2: Standardized Differences before and after IPTW; Figure S3: Kaplan–Meier curve before IPTW; Table S1: Numbers of Implants during the observation period; Table S2: Numerical distribution of TF-TAVI and TA-TAVI per year; Table S3: Preoperative medications.

## Abbreviations

CABG	Coronary Artery Bypass Grafting
CAD	Coronary Artery Disease
CI	Confidence Interval
CT	Computer Tomography
COPD	Chronic Obstructive Pulmonary Disease
HR	Hazard Ratio
IPTW	Inverse probability of treatment weighting
MI	Myocardial Infarction
PAD	Peripheral Artery Disease
PM	Pacemaker
SAVR	Surgical Aortic Valve Replacement
STS	Society of Thoracic Surgeons
TA	Transapical
TAVI	Transcatheter Aortic Valve Implantation
TF	Transfemoral
VARC	Valve Aortic Research Consortium
