# Supplementary material for: Similar 5-Year Survival in Transfemoral and Transapical TAVI Patients: A Single-Center Experience
Source: Bioengineering (Basel). 2023 Jan 24;10(2):156. doi: 10.3390/bioengineering10020156 (PMC9952102; doi:10.3390/bioengineering10020156)
Supplement: Supplementary file 1 [file bioengineering-10-00156-s001.zip › bioengineering-2159589-supplementary.pdf]

Supplementary Materials

Similar 5-Year Survival in Transfemoral and Transapical TAVI Patients: A Single-Center Experience

Constantin Mork [1,2,†], Raphael Twerenbold [1,3,4,†], Brigitta Gahl [2], Friedrich Eckstein [2], Raban Jeger [1,5], Christoph Kaiser [1,6] and Oliver Reuthebuch [2,*]

1 Department of Cardiovascular Research Institute Basel (CRIB), University Hospital Basel, 4031 Basel, Switzerland
2 Department of Cardiac Surgery, University Hospital Basel, 4031 Basel, Switzerland
3 University Center of Cardiovascular Science, University Heart and Vascular Center Hamburg, 20251 Hamburg, Germany
4 Department of Cardiology, University Heart and Vascular Center Hamburg, 20251 Hamburg, Germany
5 Department of Cardiology, Triemli Hospital Zurich, 8063 Zurich, Switzerland
6 Department of Cardiology, University Hospital Basel, 4031 Basel, Switzerland
* Correspondence: oliver.reuthebuch@usb.ch
† Both authors should be considered as first shared authors.

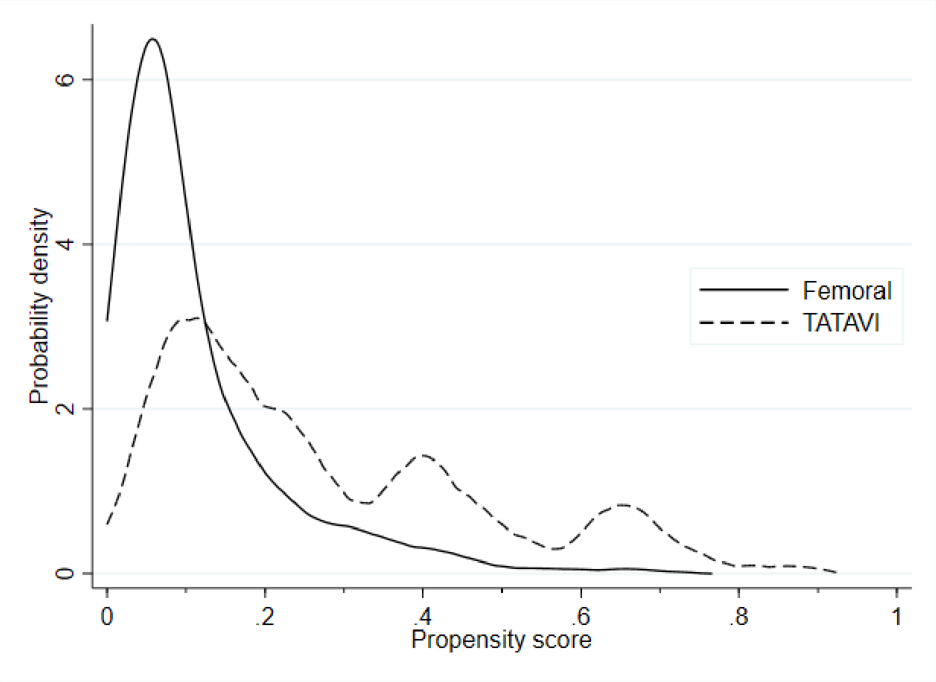

Figure S1. Propensity Score, kernel density.

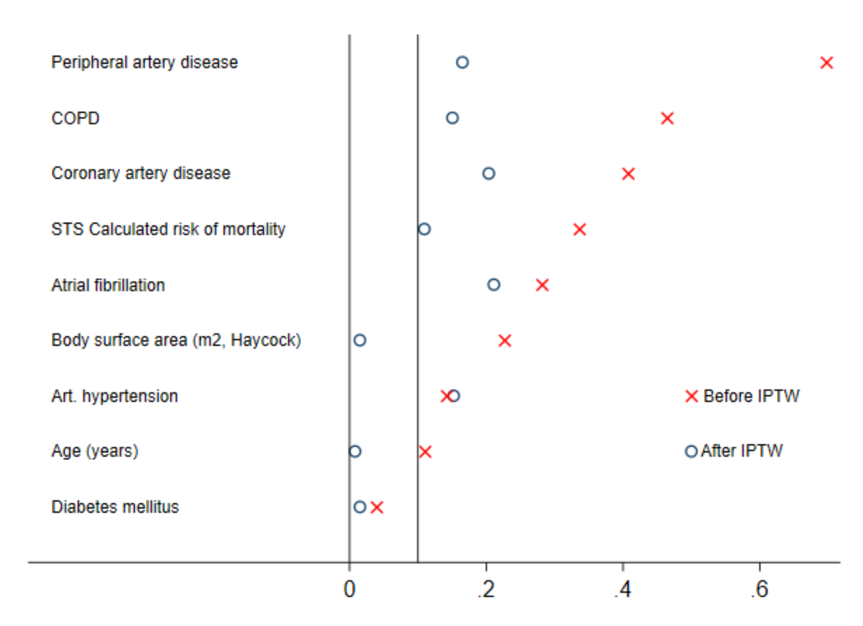

Figure S2. Standardized Differences before and after IPTW.

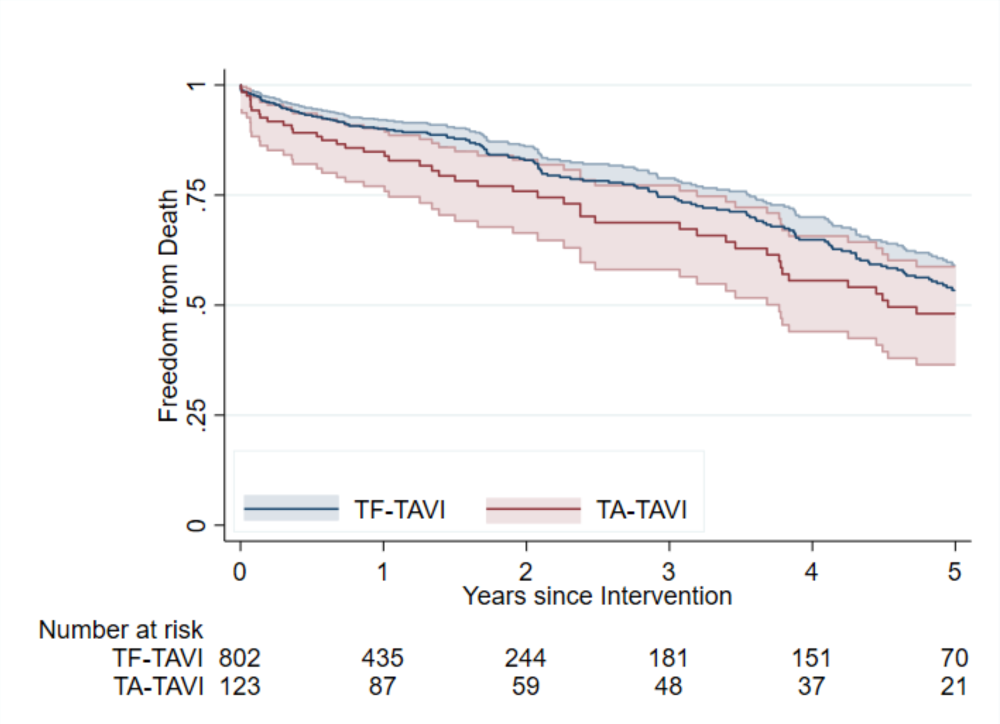

Figure S3. Kaplan-Meier curve before IPTW.

Table S1. Numbers of Implants during the observation period.

	TF-TAVI	TA-TAVI
	N = 802	N = 123
Device		
Medtronic CoreValve [NO LONGER USED]	69 (8.6%)	0 (0.00%)
Edwards Sapien XT [NO LONGER USED]	69 (8.6%)	9 (7.3%)
Symetis Acurate / - NEO	137 (17%)	2 (1.6%)
JenaValve	1 (0.12%)	56 (46%)
SJM Portico	177 (22%)	0 (0.00%)
Medtronic Engager [NO LONGER USED]	0 (0.00%)	2 (1.6%)
Direct Flow Medical	5 (0.62%)	0 (0.00%)
Edwards Sapien 3	155 (19%)	53 (43%)

BSC Lotus [NO LONGER USED]	68 (8.5%)	0 (0.00%)
Medtronic Evolut R	60 (7.5%)	0 (0.00%)
Lotus Edge	19 (2.4%)	0 (0.00%)
Evolut PRO	38 (4.7%)	1 (0.81%)

TF-TAVI: transfemoral transcatheter aortic valve implantation; TA-TAVI: transapical transcatheter aortic valve implantation.

Table S2. Numerical distribution of TF-TAVI and TA-TAVI per year.

**Numerical distribution per year**	**TF-TAVI,**	**TA-TAVI**
	N = 802	N = 123
2011, n = 10	80.0	20.0
2012, n = 65	67.7	32.3
2013, n = 85	77.6	22.4
2014, n = 93	75.3	24.7
2015, n = 96	84.4	15.6
2016, n = 122	86.1	13.9
2017, n = 108	91.7	8.3
2018, n = 105	95.2	4.8
2019, n = 144	95.1	4.9

Table S3. Preoperative medications

	**TF-TAVI**	**TA-TAVI**	**p**
	**N = 802**	**N = 123**	
Aspirin	433 (54%)	90 (73%)	<0.001
P2Y12 antagonist	163 (20%)	27 (22%)	0.72
Clopidogrel	147 (18%)	23 (19%)	0.9
Prasugrel	1 (0.12%)	2 (1.6%)	0.048
Ticagrelor	15 (1.9%)	2 (1.6%)	1
Marcoumar/Sintrom	162 (20%)	22 (18%)	0.63
Novel Anticoagulation drugs	125 (16%)	11 (8.9%)	0.06

TF-TAVI: transfemoral transcatheter aortic valve implantation; TA-TAVI: transapical transcatheter aortic valve implantation.
